# Anti-angiogenesis therapy based on the bone marrow-derived stromal cells genetically engineered to express sFlt-1 in mouse tumor model

**DOI:** 10.1186/1471-2407-8-306

**Published:** 2008-10-23

**Authors:** M Hu, J-L Yang, H Teng, Y-Q Jia, R Wang, X-W Zhang, Y Wu, Y Luo, X-C Chen, R Zhang, L Tian, X Zhao, Y-Q Wei

**Affiliations:** 1 State Key Laboratory of Biotherapy, West China Hospital, Sichuan University, Chengdu 610041 Sichuan, PR China; 2 The Central Laboratory, West China Second University Hospital, West China Medical School, Sichuan University, Chengdu, 610041, Sichuan, PR China; 3 Department of respiratory medicine, People's Hospital of Sichuan Province, Chengdu, 611830, Sichuan, PR China

## Abstract

**Background:**

Bone marrow-derived stromal cells (BMSCs) are important for development, tissue cell replenishment, and wound healing in physiological and pathological conditions. BMSCs were found to preferably reach sites undergoing the process of cell proliferation, such as wound and tumor, suggesting that BMSCs may be used as a vehicle for gene therapy of tumor.

**Methods:**

Mouse BMSCs were loaded with recombinant adenoviruses which express soluble Vascular Endothelial Growth Factor Receptor-1 (sFlt-1). The anti-angiogenesis of sFlt-1 in BMSCs was determined using endothelial cells proliferation inhibition assay and alginate encapsulation assay. The anti-tumor effects of BMSCs expressing sFlt-1 through tail-vein infusion were evaluated in two mouse tumor metastases models.

**Results:**

BMSCs genetically modified with Adv-GFP-sFlt-1 could effectively express and secret sFlt-1. BMSCs loaded with sFlt-1 gene could preferentially home to tumor loci and decrease lung metastases and prolong lifespan in mouse tumor model through inducing anti-angiogenesis and apoptosis in tumors.

**Conclusion:**

We demonstrated that BMSCs might be employed as a promising vehicle for tumor gene therapy which can effectively not only improve the concentration of anticancer therapeutics in tumors, but also modify the tumor microenvironment.

## Background

Bone marrow-derived stromal cells (BMSCs), also known as mesenchymal stem cells or nonhematopoietic progenitor cells, are precursors that can be differentiated into chondrocytes, osteoblasts, adipocytes, neurons and other cell types [1]. They are important for development and cell replenishment of active proliferating tissues in physiological conditions, such as blood, skin and gut. In pathological conditions, they are involved in the process of wound healing and tissue regeneration. In animal and clinical experiments, BMSCs have been used for tissue damage repair and functional reconstruction of organs, such as myocardial infarction, osteogenesis imperfecta, syndrome of multiple organ failure and rebuilding of hematopoietic system after chemotherapy of breast cancer [2-5].

It has been reported that the conditions featured by enhanced cell proliferation and tissue remodeling, such as bone fractures, embryo growth and tumorigenesis, offer an appropriate microenvironment for migration, proliferation and differentiation of stem cells delivered systemically [3,6-9]. The formation of tumor stroma is similar to that of wound healing and results in a microenvironment which is suitable for the proliferation of bone marrow-derived stem cells [10]. Therefore, we hypothesize that systemically delivered BMSCs would preferentially home to tumor tissues and participate in the formation of tumor stroma. Therefore, it will be useful to develop a new targeting system based on genetically modified BMSCs for tumor gene therapy. The feasibility of this approach has been demonstrated by Matus Studeny's experimemt [11].

Metastasis is the most common and fundamental characteristics of solid tumors. Most deaths of cancerous patients result from the ruthless growth of metastases that are resistant to conventional remedies. This urges us to develop a novel therapeutic strategy for inhibiting tumor metastasis. The concept that tumor growth and metastasis relies on angiogenesis has been widely proven and accepted. The signal axis of vascular endothelial growth factor (VEGF) and its receptors (VEGFR-1, VEGFR-2 and VEGFR-3) is involved in the formation and progress of tumors. The soluble VEGF receptor-1 (sFlt-1) has been shown to be effective in inhibition of cancerous angiogenesis [12].

In this report, we developed a novel strategy of tumor gene therapy in which BMSCs loaded with recombinant adenoviruses expressing sFlt-1 could effectively suppress tumor growth through inhibiting angiogenesis and metastases and prolong the lifespan in mouse model, indicating that BMSCs might be employed as an effective carrier for tumor gene therapy.

## Methods

### BMSCs isolation and culture

BMSCs were isolated from femur bone marrow of male BALB/c mouse. Mononuclear cells were separated by centrifugation over a Ficoll gradient (Sigma Chemical Co., St. Louis, MO) of 1.077 g/ml. The cells were cultured in L-DMEM containing 10% fetal bovine serum (Gibco BRL, Inc) at an initial seeding density of 1 × 10^5 ^cells/cm^2 ^at 37°C with 5% CO2 and 95% humidified atmosphere. The nonadherent cells were removed and washed with PBS after 24 hours, and the monolayer of adherent cells were cultured until they reached to confluence. The cells were then trypsinized (0.25% trypsin with 0.1% EDTA), subcultured at densities of 5000–6000 cells/cm^2. ^and used for experiments during passages five to eight. CD34, CD29 and CD44 expressions in BMSCs were determined by flow cytometry. Briefly, the trypsinized BMSCs were adjusted to 1 × 10^7^cells/ml in media (1% bovine serum albumin, 0.2% sodium azide in PBS), and then stained with FITC-conjugated monoclonal antibodies (BD Biosciences, San Jose, CA, USA) on ice according to the manufacturer's protocol, and followed by detection with flow cytometry.

### Production of adenovirus and genetic modification of BMSCs

AdenoVec-GFP-sFlt-1 and AdenoVec-GFP (InvivoGen, San Diego, CA) were transfected into HEK-293 cells using jetPEI (Qbiogene, Nottingham, UK) according to the manufacturer's protocol. Seven to 14 days later, the recombinant adenoviruses were harvested from the cultures and the viruses were further amplified in 293 cells, and then tittered by plaque assay. BMSCs were infected with adenoviruses at a multiplicity of infection (MOI) of 3000 for 2 hours followed by replacing the infection medium. Twenty-four hours later, the virus-infected BMSCs were harvested for subsequent experiments [11].

### Western blot analysis

Western blot analysis was performed as described previously [13]. Briefly, the secreted proteins in supernatants of the culture were precipitated by TCA-DOC/acetone and the western blot assay was performed using standard method with primary monoclonal antibody of anti-sFlt-1 (1:500 dilution, Santa Cruz Biotechnology, Santa Cruz, CA, USA) and a biotinylated secondary antibody, and the proteins on the membrane were visualized by Vectastain ABC kit (Vector Laboratories, Burlingame, CA, USA).

### Mouse endothelial cells proliferation inhibition assay

The mouse endothelial cells proliferation inhibition assay was performed as described previously for examining suppression of sFlt-1 in the conditioned media on VEGF-driven proliferation of endothelial cells [14]. The conditioned media were obtained from BMSCs infected with Adv-GFP-sFlt-1, Adv-GFP, or PBS (mock infected), respectively. Endothelial cells were grown in 24-well plates at 37°C in DMEM containing 10% FBS. At 50% confluence, the cells were washed with PBS after removal of the DMEM media, and then 0.5 ml of conditioned media was added. Fifteen minutes later, VEGF was added to each well with a final concentration of 10 ng/ml and the cells were incubated at 37°C. After 72 h, the cells were trypsinized, and the number of viable cells was counted using a trypan blue assay.

### Alginate encapsulation assay

Alginate-encapsulated tumor cell assays were performed as described previously [13]. Briefly, tumor cells, BMSCs expressing sFlt-1 or mixed cells were resuspended in a 1.5% solution of sodium alginate and added dropwise into a swirling 37°C solution of 250 mM calcium chloride. Alginate beads were formed with about 1 × 10^5 ^cells per bead. After mice were anesthetized, 4 beads were implanted subcutaneously into an incision made on the dorsal side. Incisions were closed with surgical clamps. After 21 days, mice were injected intravenously with 100 μl of a 100 mg/kg FITC-dextran solution (Sigma). Beads were surgically removed, and FITC-dextran was quantified against a standard curve of FITC-dextran.

### Murine tumor metastases models and treatment

The colon carcinoma CT26, Lewis lung cancer LLC and fibrosarcoma MethA cell lines were used in this study. Metastasis models of LLC and CT26 were generated as described previously [15]. Briefly, female C57BL/6 and BALB/c mice were received i.m. injections of 2×10^5 ^LLC in leg or i.v. injection of 2×10^5 ^CT26 cells from tail vein in 100 μl of PBS, respectively and then the animals were randomly divided into four groups (10 mice/group). After 4 days for CT26 model and 10 days for LLC model of tumor inoculation, 1×10^6 ^of BMSCs infected with Adv-GFP-sFlt-1, BMSCs infected with Adv-GFP, unifected BMSCs or 100 μl of 0.9% NaCl solution alone were administered via tail vein for four doses at 3 days intervals for CT26 model and 5 days intervals for LLC model. Mice received LLC or CT26 injection were sacrificed on day 32 or day 16 after inoculation, respectively, when control mice became moribund, and the lungs from each mouse were removed and the surface lung metastases (>3 mm) were measured and scored [15]. The lungs of animals were also fixed in 10% buffered formalin followed by histological analysis. All of the mice used in this study were approved by the West China Hospital Cancer Center's Animal Care and Use Committee. In the handling and care of animals, all possible steps were taken to avoid animals' suffering and efforts were made to use the minimum number of animals.

### Histological analysis

The tissues were fixed in 10% neutral buffered formalin solution and embedded in paraffin. Sections of 3–5 μm were stained with hematoxylin and eosin (H.E.). Frozen sections were fixed in acetone, incubated, and stained with anti-CD31 antibody, then visualized with DAKO LSAB kit (DAKO, Carpinteria, CA), as described previously. Vessel density was determined by counting the number of microvessels per high-power field in the sections, as described [16]. Apoptosis in tumor tissues was determined by terminal dUTP nick-end labeling (TUNEL) method using an *in situ *cell death detection kit (Roche Molecular Biochemicals) following the manufacturer's protocol [17,18]. Sections in H&E staining and immunohistochemical staining were observed by two pathologists in a blinded manner.

### Fluorescent in situ hybridization (FISH)

Fluorescent in situ hybridization was performed according to the mouse Y chromosome probe manual (STAR*FISH; Cambio, Cambridge, England) with some modifications. Cryostat sections of 5 μm in thickness were fixed in Carnoy's fixative for three times, 10 min each, and the sections were incubated in pepsin solution (1% in 0.1N HCl) for 10 minutes and rinsed in PBS. Serial ethanol dehydration was done (1.5 min each), and the slides were air-dried at room temperature. Sections were denatured at 65°C for 2 min in preheated 70% formamide and 2×SSC buffer, pH 7.0, and were then 'quenched' with ice-cold 70% ethanol for 1.5 min. Serial ethanol dehydration was done again. The mouse Y chromosome probe labeled with Cy3 was denatured at 65°C for 10 min and applied to the sections. The sections were coverslipped and sealed with rubber cement for incubation overnight in a hydrated slide box at 37°C. The next day, the coverslips were carefully removed. The sections were washed twice in preheated 50% formamide in 2×SSC buffer for 5 min each at 45°C and were then gently washed twice in preheated 1×SSC buffer for 5 min each at 45°C.

### Statistical assay

Significance was determined using one-way ANOVA and log-rank test (SPSS 11.0 for windows). Difference between groups were significant at a value of *P *< 0.05.

## Results

### BMSCs isolation, genetic modification and sFlt-1 function validation

**Figure 1:**
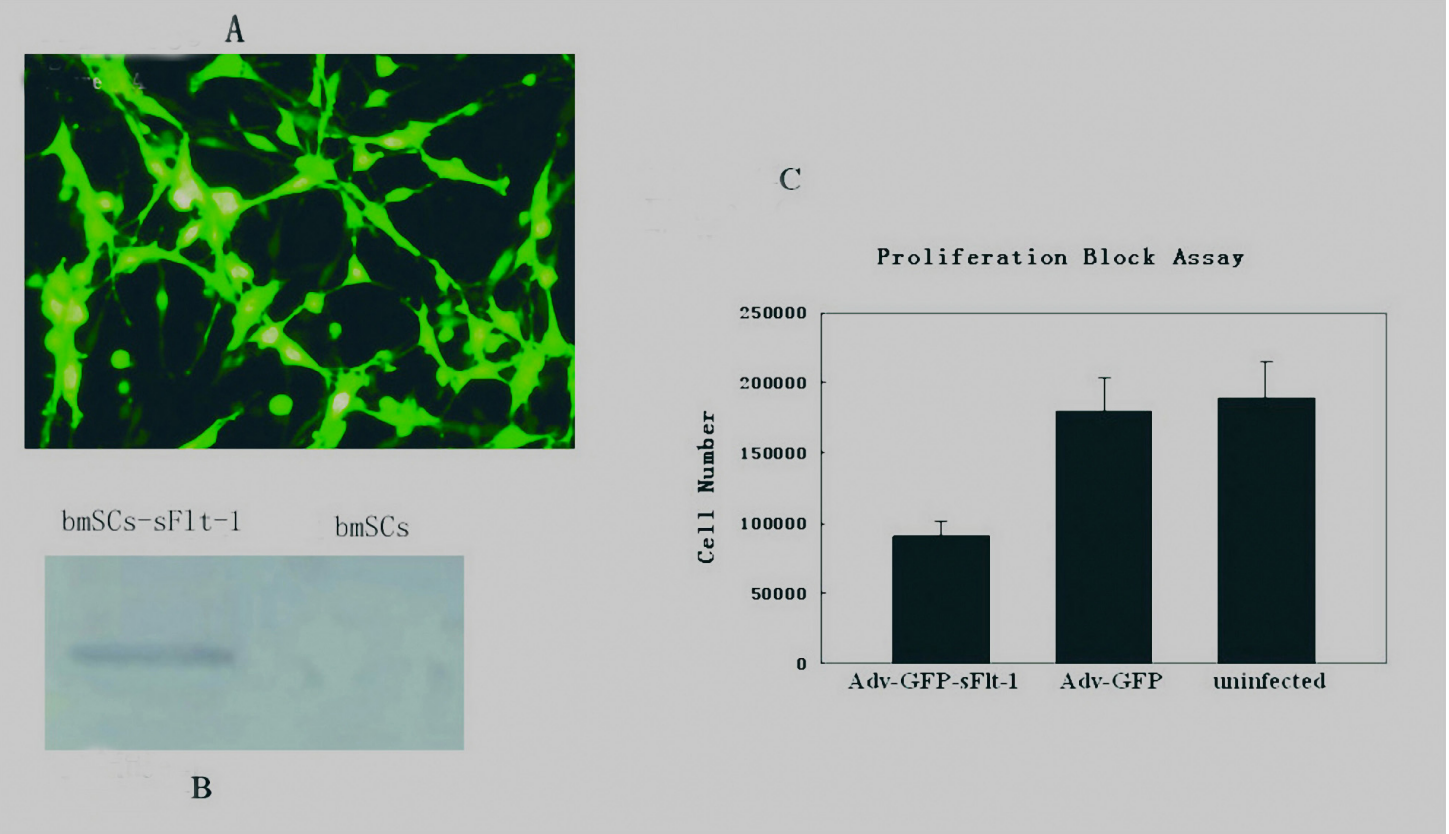
**BMSCs infection and verification of secreted sFlt-1**. After the BMSCs were confirmed by flow cytometric analysis, BMSCs were grown to 90% confluence and infected with adenoviruses at a multiple of infection of 3000 for 2 hours. 24 hours later, BMSCs were harvested and ready for use. Fluorescence microscope showed up to 100% GFP-positive cells. (Figure 1-A). Supernatants deposits from sFlt-1 bearing BMSCs could be recognized by antibodies reactive to NH_2 _terminus of mouse Flt-1, but negative staining in supernatants deposits from control BMSCs in Western blot analysis. (Figure 1-B). Conditioned media from Adv-sFlt-1 infected BMSCs was shown to inhibit the VEGF-driven mouse endothelial cells proliferation by about 50% compared with controls (Figure 1-C, P < 0.05).

After harvest of monolayer cells with spindle-like morphology from mouse bone marrow, three CD markers (CD34, CD29 and CD44) were used for characterizing BMSCs. The cells were positive for CD29, CD44, and negative for CD34, indicating that they didn't belong to hematopoietic progenitor cells, but rather bone marrow-derived stromal cells (BMSCs) or mesenchymal stem cells (MSCs). BMSCs were cultured to reach to 90% confluence and incubated with adenoviruses at a MOI of 3000 for 2 hours. Twenty four hours later, all of the cells were GFP-positive checked by fluorescence microscopy and the cells were ready for tail vein infusion after PBS washing two times (Figure 1A). The secreted sFlt-1 in the supernatants of conditioned media was verified by Western blot (Figure 1B) and the function of sFlt-1 was validated by endothelial cell growth inhibition assay *in vitro*. The concentrated conditioned media obtained from BMSCs infected with Adv-GFP-sFlt-1 or with Adv-GFP were applied to the mouse endothelial cells grown in 24-well plates, followed by stimulation with a 10 ng/ml of VEGF 15 minutes later. Conditioned media from BMSCs infected with Adv-GFP-sFlt-1 resulted in inhibition of endothelial cells proliferation by about 50% compared with that from BMSCs infected with Adv-GFP or uninfected BMSCs (Figure 1C, P < 0.05), indicating that the BMSCs infected with Adv-GFP-sFlt-1 could effectively express and secret sFlt-1.

### Anti-metastasis effect

**Figure 2:**
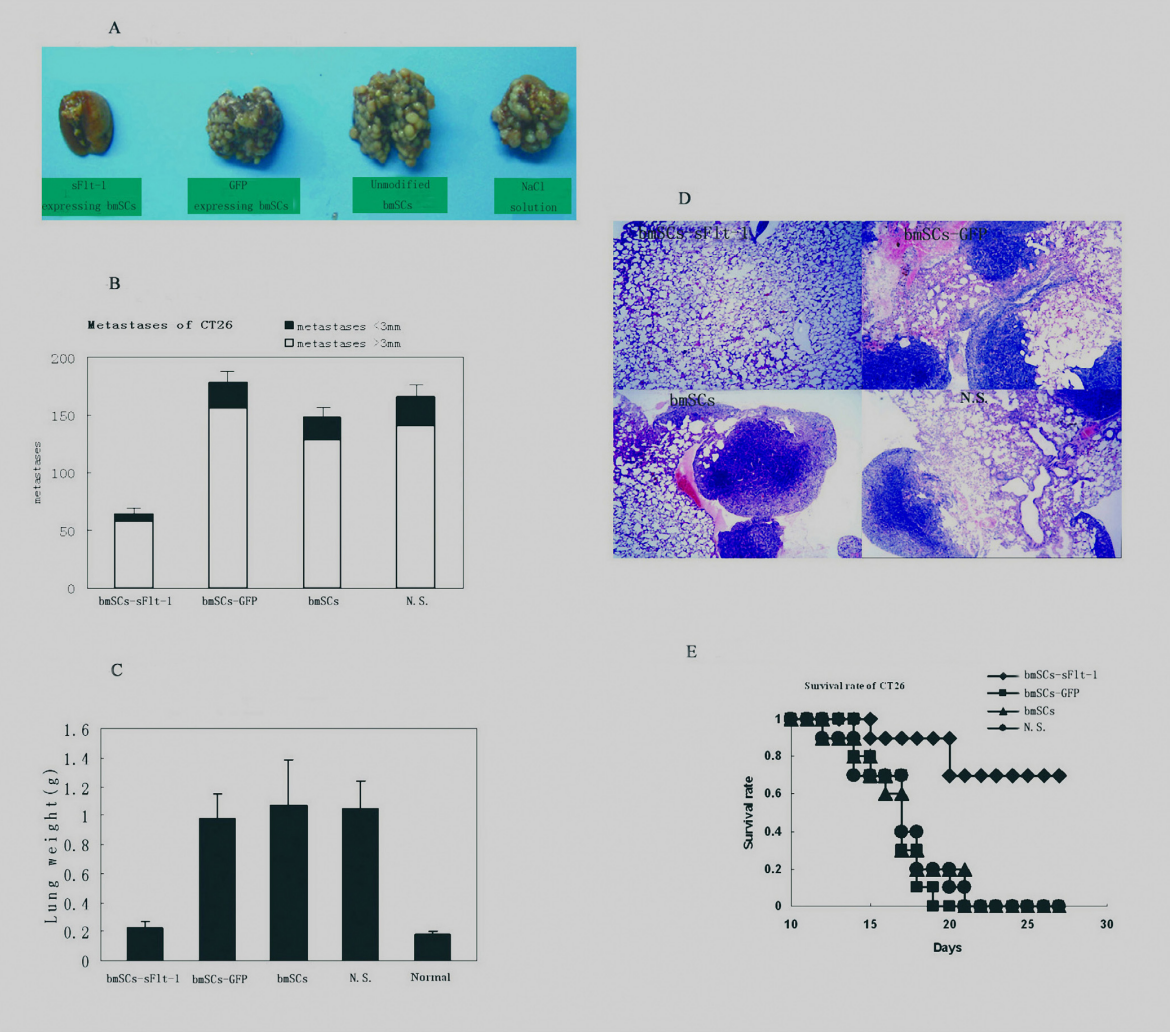
**Anti-tumor effect in mouse CT26 colon adenocarcinoma model**. Female BALB/c mice received i.v. injection of 2×10^5 ^CT26 cells in 100 μl of PBS. Treatment with 1×10^6 ^Adv-GFP-sFlt-1 infected BMSCs(◆, black rhombus), 1×10^6 ^Adv-GFP infected BMSCs (▪, black quadrilateral), 1×10^6 ^uninfected BMSCs (▲, black triangle) and 100 μl of 0.9% NaCl solution alone (●, black circularity) were administered i.v. from tail vein. Treatment with sFlt-1-bearing BMSCs could decrease the number of and growth of surface metastases (Figure 2-A, D) and abolish the tumor burden by rendering the weight of lungs similar to that of normal mice (Figure 2-C). Mean numbers of lung tumor nodules in each group are shown (Figure 2-B). Values are plotted as means ± SEM (P < 0.05). The percentage of metastatic foci > 3 mm are marked as solid bars. A significant increase of survival rate in sFlt-1-bearing BMSCs treated mice, compared with the controls (Figure 2-E, P < 0.05. By log-rank test), was found in the tumor model.

**Figure 3:**
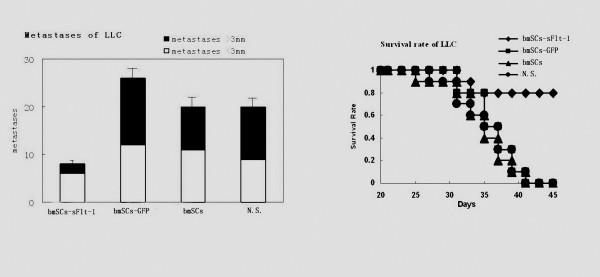
**Anti-tumor effect in mouse LLC lung carcinoma model**. Female C57BL/6 mice received i.v. injection of 2×10^5 ^LLC cells in 100 μl of PBS. Treatment with 1×10^6 ^Adv-GFP-sFlt-1 infected BmSCs (◆, black rhombus), 1×10^6 ^Adv-GFP infected BmSCs (▪, black quadrilateral), 1×10^6 ^uninfected BmSCs (▲, black triangle) and 100 μl of 0.9% NaCl solution alone (●, black circularity) were administered i.v. from tail vein. A significant increase of survival and decrease of metastases in sFlt-1-expressing BMSCs treated mice, compared with the controls (P < 0.05. By log-rank test), was found in this model.

The mouse CT26 and LLC metastasis models were used to determine whether BMSCs expressing sFlt-1 suppress the growth of metastatic neoplasm. The metastatic nodules > 3 mm were assessed as an indicator of angiogenesis because the growth of tumors > 3 mm is thought to be vasculature sensititive [19]. Most of the mice systemically administered with control BMSCs or NaCl solution developed macroscopic lung metastases, whereas mice treated with BMSCs expressing sFlt-1 decreased lung metastases. The tumor nodules (> 3 mm) of lung surface were significantly decreased in the mice treated with BMSCs expressing sFlt-1 (*P *< 0.05) (Figure 2A, B). Lung weight, which correlates with total tumor burden, was also reduced significantly in the team of mice administered with BMSCs expressing sFlt-1 (Figure 2C). Booming proliferation of tumor cells and severe destruction of lung tissues were observed in the H.E. staining for control mice, whereas in the mice treated with BMSCs expressing sFlt-1, cancerous cells were less nourished and lung tissues were relatively normal (Figure 2D). Benefited from the decrease of tumor burden in the lung metastases, the lifespan of the tumor-bearing mice was prolonged significantly (Figure 2E, P < 0.05). The similar results were also observed from mouse LLC metastasis model (Figure 3).

### Inhibition of angiogenesis and induction of apoptosis

**Figure 4:**
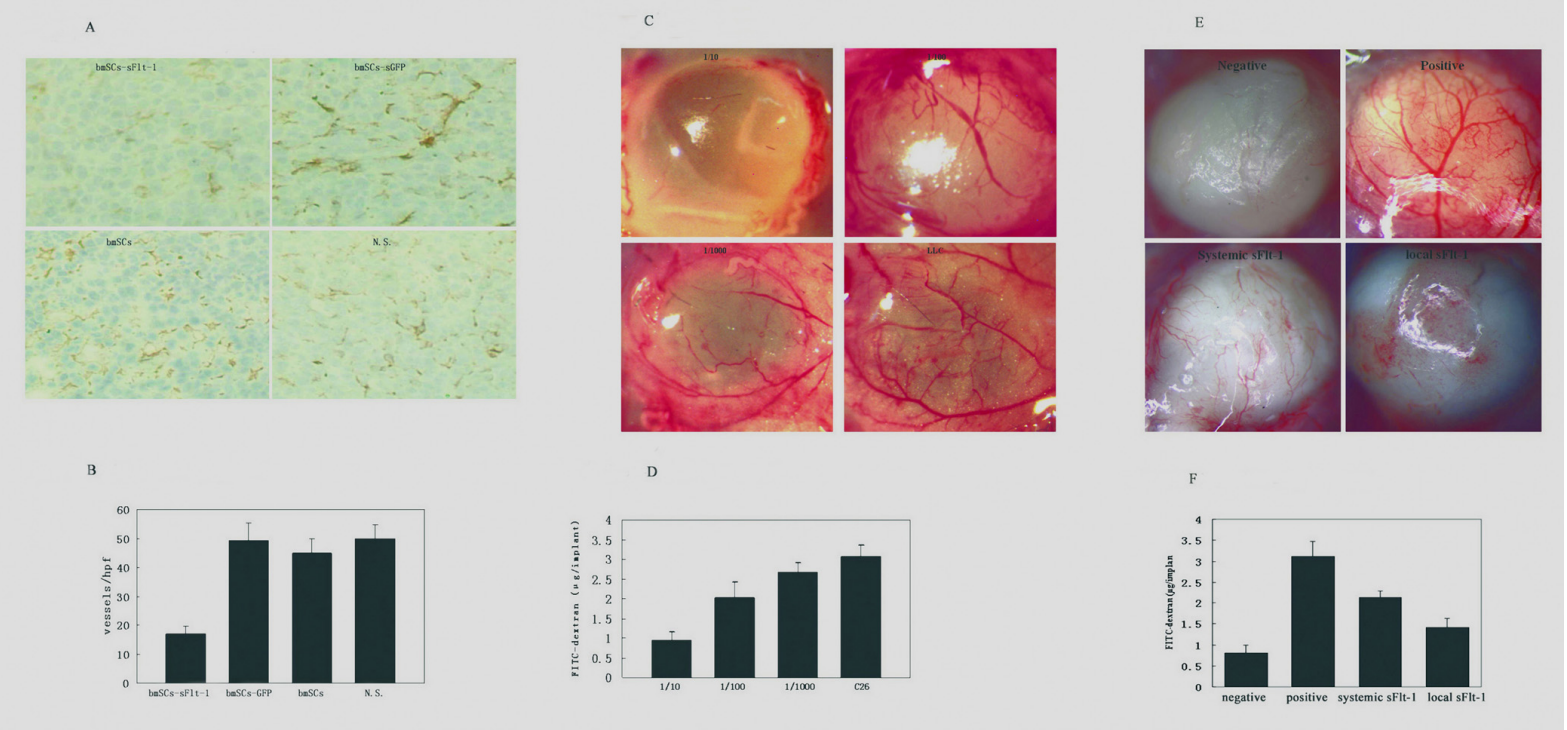
**Evaluation of anti-angiogenetic effect by CD31 immunohistochemistry and alginate assay**. Sections of frozen CT26 tumor tissues obtained from mice treated with sFlt-1-bearing BMSCs, GFP-expressing BMSCs, unmodified BmSCs and 0.9% NaCl solution were stained with CD31 antibody and Peroxidase-DAB (Figure 4-A). Vessel density was determined by counting the number of microvessels per high-power field (×200) in sections. (Figure4-B). Alginate beads containing different ratio of sFlt-1-bearing BMSCs and LLC (1/10, 1/100, 1/1000, LLC only) were implanted subcutaneously into C57BL/6 mice. Three weeks later, beads were surgically removed, and FITC-dextran was quantified. FITC-dextran uptake decrease and photograph of alginate implants showed the reduction of vascularization in beads containing relatively more sFlt-1-expressing BMSCs. (Figure4-C and D). Alginate beads containing MethA, BMSCs and mixture of both with ratio of 1/10 were prepared. Beads-MethA and beads-mixture were served as positive and negative controls. The third team of mice was implanted with two different beads containing MethA or BMSCs respectively in different sites of mice, which served as systemic sFlt-1 model to show the non-targeted effect of viruses. The fourth team of mice was implanted with beads containing MethA and hence injected intravenously with BMSCs in the same number of the former team, which served as local sFlt-1 model to show the targeted effect of viruses loaded in BMSCs. Compared with the controls, the effect of systemic production of sFlt-1 was weaker than local one. (Figure 4-E and F).

**Figure 5:**
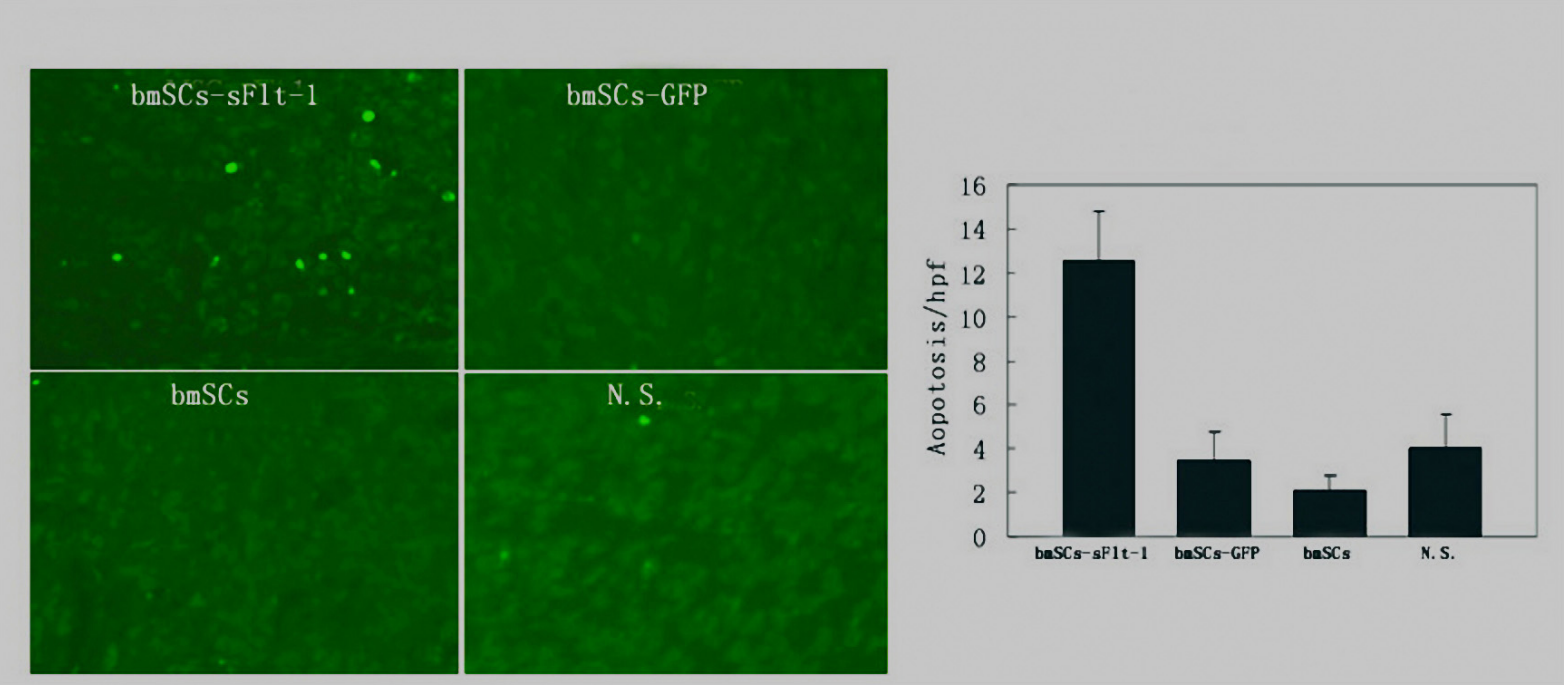
**Apoptosis Assay by TUNEL staining**. Paraffin sections from CT26 tumor tissues obtained from mice systemically administered with sFlt-1-expressing BMSCs, GFP expressing BMSCs, unmodified BMSCs and NaCl solution were stained using an *in situ *cell death detection kit (Roche) following the manufacturer's protocol. Apoptosis was determined by counting the number of the positive cells per high-power field in tumor sections. Original magnification, ×200. (Figure 5-A and B, P < 0.05).

Systemically delivered BMSCs which express sFlt-1 were shown to induce apparent inhibition of angiogenesis *in vivo *compared with control mice by immunohistochemistry. Angiogenesis within tumor tissue was estimated by counting the number of microvessels on the section staining with an anti-CD31 antibody. Very few newborn microvessels were found around the tumor cells in the BMSCs-sFlt-1 group, whereas abundant microvessels existed in three controls (Figure 4A, B, P < 0.05). The inhibition of angiogenesis in mice treated with BMSCs expressing sFlt-1 was further confirmed in alginate encapsulation assay. The surface vessel densities and the FITC-dextran uptake were apparently reduced in beads which contain relatively high ratio of sFlt-1-bearing BMSCs to LLC cells (Figure 4C, D, P < 0.05). Furthermore, an another alginate assay showed that the anti-angiogenesis effect of local production of sFlt-1 (came from the alginate beads being filled with sFlt-1-bearing BMSCs and tumor cells, or the BMSCs intravenous injected) was stronger than systemic production of sFlt-1 (came from the alginate beads being filled with sFlt-1-bearing BMSCs and implanted in the distant site) (Figure 4-E and 4F). Being starved of the nourishment by the inhibition of angiogenesis in neoplasm, augmentation of apoptosis of cancerous cells was detected by the TUNEL assay (Figure 5, P < 0.05). The inhibition of angiogenesis and the consequent induction of apoptosis underlie the mechanism of the metastases decrease and the life-span prolongation in tumor-bearing mice.

### Verification of BMSCs' homing to tumor

**Figure 6:**
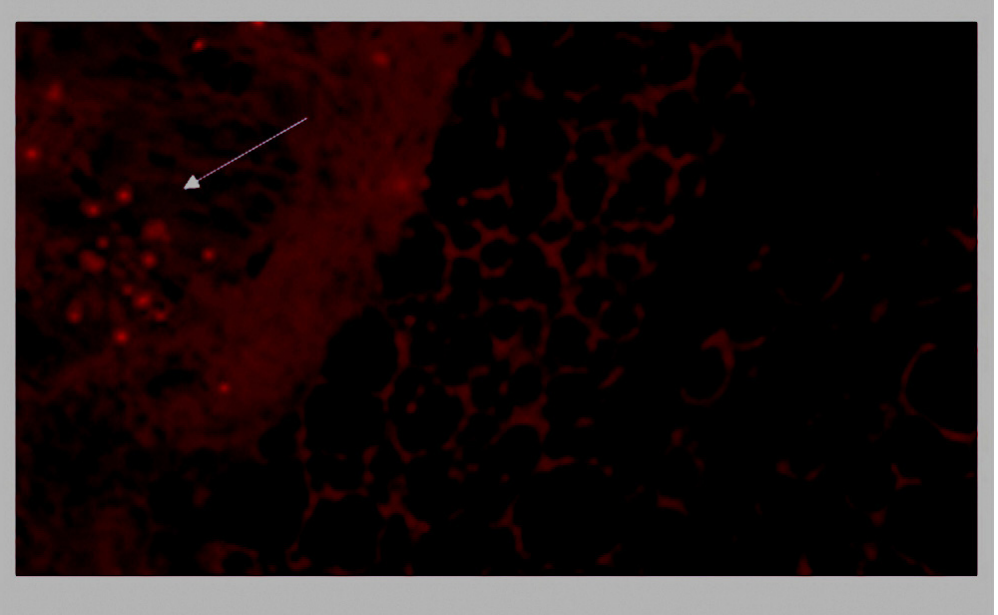
**Fluorescent in situ hybridization**. Frozen sections of lung metastases were tested for the presence of Y chromosome which is indication of male originated BMSCs by Cy3 labeled Y chromosome probe (STAR*FISH; Cambio, Cambridge, England). Positive result could be seen in the tumor area obviously, whereas the relatively normal parts of the sections were totally negative for fluorescent signals.

It has been reported that ubiquitous tissue distribution of adipose-derived stem cells in normal mouse was observed when the cells were systematically administrated [20]. We are concerned about the existence of BMSCs in the metastases and the surrounding tissues. To detect the tumor tropism of BMSCs after systemically administration, fluorescent *in situ *hybridization of Y chromosome was performed on frozen section of CT26 lung metastasis after BMSCs administration one week. Positive signals were observed in the frozen section of lung metastases exclusively, whereas the relatively normal area of lung tissue was totally negative for the fluorescent signals (Figure 6). The tropism of BMSCs to the sites undergoing active cell growth and differentiation makes them a smart delivery system of therapeutic agents, especially in gene therapy of cancer and severe destruction of tissues.

## Discussion and conclusion

BMSCs have been used in the therapy of myocardial infarction, osteogenesis imperfecta, syndrome of multiple organ failure and rebuilding of haematopoietic function after chemotherapy of breast cancer in pre-clinical and clinical experiments [2-5]. In this report, we demonstrated that systemically delivered BMSCs loaded with sFlt-1 gene could preferentially home to tumor loci and induce anti-angiogenesis as well as apoptosis of cancerous cells, resulting in the decrease of lung metastases and prolongation of lifespan in mouse tumor model.

The BMSCs' capability of tissue repairing and homing has been attracted great attention of scientists. Grove reported that up to 20% of lung epithelium could be derived from circulating BMSCs after lung injury, and a large portion of lung damage could be repaired by forming entire alveoli. Meantime, these adult stem cells could be effectively used to deliver therapeutic gene to lung while retaining the ability to differentiate into lung cells [21]. Darwin J Prockpop demonstrated that injured tissue create a milieu that enhances homing, proliferation and differentiation of BMSCs [7]. It has been found that there are high concentrations of fibroblast growth factor, transforming growth factor β, platelet-derived growth factor, and epidermal growth factor in injured tissue and in the microenvironment of tumor, in which these facters are needed in the process of homing, proliferation and differentiation of BMSCs [22,23]. Therefore, we hypothesize that tissue rebuilding in tumorigenesis, wound healing and fetal development entitles the microenvironments the capability of attracting the systemically administered BMSCs.

Suppression of VEGF by sFlt-1 adenoviruses administered systemically was proved to contribute to inhibition of tumor growth [12]. In our study, we employed BMSCs as a gene vehicle loaded with sflt-1 recombinant adenovirus for tumor therapy, and found that the BMSCs expressing sFlt-1 preferentially homed to and survived in the tumor loci where its strong anti-angiogenesis and anti-metastasis effect has been observed. We also observed that BMSCs expressing IL-12 induced a stronger antitumor effect than administration with IL-12 adenoviruses alone (data not shown). This vehicle may be useful and effective in tumor gene therapy for four reasons. Firstly, the BMSCs after ex vivo administration may survive for hundreds of days, which enable them to offer a relatively long effect for chronic diseases [24]. Secondly, the capacity of BMSCs homing to tumor sites and protecting viruses against the immune surveillance would strengthen the effect of recombinant adenovirus gene therapy, which is unsatisfactory when systemically administered alone because of nonspecific distribution and serious hepatotoxicity [11,25]. Thirdly, the lower immunogenicity, which is the result of low expression of MHC and costimulatory molecules, enables BMSCs advantageous for allogeneic or xenogeneic applications [26]. Last, BMSCs are easy to be expanded *in vitro *and to be genetically modified, which means convenience and cost effectiveness in the future preclinical or clinical applications.

However, in the experiments performed by our lab (data not shown) and others, the naive BMSCs seemed to promote growth of tumors. On the other hand, Aarif reported that mesenchymal stem cells injected intravenously homed to neoplasm and potently inhibited tumor growth in Kaposi sarcoma model [27]. The relationship between the stem cells and the cancer cells may depend on the microenvironment which is created by these two kinds of cells and others [28-31]. Besides, the possible transformation and the consequent tumorigenesis of BMSCs themselves remains to be clarified. Jakub and his colleagues reported that bone marrow-derived mesenchymal stem cells showed cytogenetic aberrations after several passages *in vitro*, which resulted in malignant lesions when infused to secondary recipients [32,33]. All of these contradictions indicated that the fate and effect of BMSCs on tumor growth should be further investigated.

In conclusion, we demonstrated that BMSCs might be employed as a promising vehicle for tumor gene therapy which can effectively not only improve the concentration of anticancer therapeutics in tumors, but also modify the tumor microenvironment. However, the fate and effect on tumor growth of BMSCs systemically administrated should be further evaluated.

## Competing interests

The authors declare that they have no competing interests

## Authors' contributions

JLY and YQW conceived of the study, and participated in its design and coordination. MH, JLY and HT participated in the design, cell experiment, animal experiment and drafted the manuscript. XZ, YQJ, LT carried out the cell culture and relevant analysis. RW, XWZ carried out the immunochemistry experiment, statistical analysis. YW, YL carried out the mouse alginate test. XCC, RZ participated in the pathologic analysis. All authors read and approved the final manuscript.

## Pre-publication history

The pre-publication history for this paper can be accessed here:


